# Potential mechanisms of endophytic *Bacillus velezensis* JR4 as a biocontrol agent against *Sclerotinia sclerotiorum* causing postharvest rot in carrot

**DOI:** 10.1186/s12866-026-05623-2

**Published:** 2026-09-28

**Authors:** Mohammed A. E. Selim, Abdelrahman M. Elattaapy, Sabrien A. Omar, Xingfeng Shao, Elsherbiny A. Elsherbiny

**Affiliations:** 1 Agricultural Microbiology Department, Faculty of Agriculture, Mansoura University, Mansoura, 35516 Egypt; 2 State Key Laboratory for Quality and Safety of Agro-products, Zhejiang Key Laboratory of Intelligent Food Logistic and Processing, College of Food Science and Engineering, Ningbo University, Ningbo, 315800 China; 3 Plant Pathology Department, Faculty of Agriculture, Mansoura University, Mansoura, 35516 Egypt

**Keywords:** Antagonistic activity, Lytic enzymes, Defense-related enzymes, Gene expression, Cytotoxicity

## Abstract

Carrot production regions worldwide incur enormous annual economic losses due to postharvest white mold caused by *Sclerotinia sclerotiorum*. Biological control is a promising approach for controlling postharvest pathogens. This study investigated the biocontrol efficacy of using *Bacillus velezensis* JR4 against Sclerotinia rot and its potential mechanisms of action. The results showed that the JR4 strain achieved 91.1% inhibition of *S. sclerotiorum* in a dual-culture assay. *B. velezensis* JR4 was able to produce extracellular enzymes such as protease, cellulase, and chitinase. Applying JR4 bacterial suspension at 1 × 10^8^ CFU mL^− 1^ on postharvest carrots infected with the pathogen reduced rot incidence by 65.4% and lesion length by 80.6% on day 5. The same concentration increased the levels of total phenolics and total flavonoids, as well as the activity of defense-related enzymes, including peroxidase (POD), polyphenol oxidase (PPO), and phenylalanine ammonia-lyase (PAL). It also increased the expression of three defense-related genes in postharvest carrots over four days. Compared to untreated carrots, it exhibited lower levels of cell membrane permeability, malondialdehyde (MDA), superoxide anion (O_2_^−^), and hydrogen peroxide (H_2_O_2_). Furthermore, the cell-free culture filtrate of JR4 showed minimal to no toxicity on WI-38 and Vero cells across all concentrations. The results suggest that *B. velezensis* JR4 could be an effective and sustainable biocontrol agent for controlling white mold in postharvest carrots. However, further safety assessments are needed for its successful application.

## Introduction

White mold, also known as Sclerotinia rot, is one of the most serious economic fungal diseases affecting carrot (*Daucus carota* L.) production regions around the world. It can cause devastating losses of 50% to 100% during postharvest storage and transportation [1]. The disease can develop in the field during the growing season, during the harvest process, or during storage [2]. *Sclerotinia sclerotiorum* is a necrotrophic ascomycete fungus and the causal agent of the disease. It damages plants by killing their cells and absorbing nutrients from dead tissues, eventually leading to the plant’s death [3]. This pathogen can infect over 700 plant species across more than 75 families [4]. *S. sclerotiorum* forms sclerotia that can survive in the soil for up to ten years under severe conditions [5].

Oxalic acid (OA) is a key factor in the pathogenicity of *S. sclerotiorum*, particularly during the initial period of plant susceptibility. It suppresses the plant’s defense mechanisms, such as chelating the Ca^2+^ from the cell wall, generating reactive oxygen species (ROS), inhibiting the oxidative burst, and inducing programmed cell death (PCD) [6, 7]. Furthermore, *S. sclerotiorum* produces various cell wall-degrading enzymes, such as proteases, cellulases, pectinases, xylanases, and glucoamylases. These enzymes help the fungus penetrate its host and degrade cell wall components [8, 9]. In this context, *S. sclerotiorum* develops a specialized structure known as an infection cushion. This structure secretes cell wall-degrading enzymes and mycotoxins that are essential for completing the infection process and play a key role in the fungus’s pathogenicity [10].

From a control perspective, the pathogenicity of *S. sclerotiorum* is a complex and dynamic process that involves external host and environmental factors. Chemical control can be effective if timed properly. Chemical fungicides such as benomyl, carbendazim, and iprodione are still commonly used to prevent and control Sclerotinia rot in carrots during both preharvest and postharvest stages [11]. However, there is currently no fungicide available on the market that specifically targets the carrot rot after harvest [12]. On the other hand, overreliance on these fungicides can cause pathogens to develop varying levels of resistance to multiple classes of fungicides [13]. More concerning still is the fact that fungicide residues in stored products pose a serious threat to human health and the environment [14]. Therefore, developing effective and viable strategies for the sustainable control of Sclerotinia rot in postharvest carrots is urgently needed. Biological control is one of the most promising eco-friendly approaches to controlling postharvest pathogens, offering an alternative to synthetic fungicides [15].

In recent years, several antagonistic bacteria capable of reducing postharvest diseases in fruits and vegetables have been identified. Most of these bacteria used as biocontrol agents belong to the families Pseudomonadaceae, Streptococcaceae, Enterobacteriaceae, Bacillaceae, and Micrococcaceae [16]. Among these, *Bacillus* species stand out for their broad application as biocontrol agents in agricultural systems [17, 18]. One notable trait of *Bacillus* is its ability to produce a variety of bioactive metabolites. For instance, up to 12% of the *B. velezensis* QST713 genome encodes pathways that produce these compounds [19]. Another interesting feature of these bacteria is their ability to combat various pathogenic bacteria and fungi with a single strain [18, 20]. The species *B. velezensis* is one of the most effective and widely used for biocontrol because it has the largest number of unique genes involved in the production of bioactive secondary metabolites [21, 22]. Examples include using *B. velezensis* NH-13–5 against *Botrytis cinerea* to control gray mold in table grapes [23], *B. velezensis* GX0002980 for controlling mango anthracnose disease caused by *Colletotrichum gloesporioides* [24], controlling pink mold rot in cherry tomatoes with *B. velezensis* ZM202201 [25], and applying *B. velezensis* L271 against *Fusarium solani* in postharvest garlic [26]. However, to our knowledge, the ability of *B. velezensis* to control Sclerotinia rot in postharvest carrots remains unexplored. To investigate this, twenty-four endophytic bacteria were isolated from the leaves of healthy cabbage plants in various fields. Among them, *B. velezensis* JR4, which showed the strongest inhibition against the pathogen in a dual culture test, was evaluated for its effectiveness in controlling white mold in carrots.

Therefore, the objective of this study was twofold. First, to evaluate the efficacy of *B. velezensis* JR4 as a biocontrol agent for controlling postharvest white mold in carrots caused by *S. sclerotiorum*. Second, to explore the underlying mechanisms behind its antagonistic action. The results provide a basis for developing and applying *B. velezensis* strain JR4 for the biological control of postharvest diseases in carrots.

## Materials and methods

### Pathogen

*S. sclerotiorum* AUMC 5400 was obtained from the Assiut University Mycological Centre (AUMC) in Egypt. The pathogen was kept on potato dextrose agar (PDA) medium. Before the experiments, the pathogen was inoculated on PDA plates and incubated at 21 °C for 2 to 4 days.

### Isolation and molecular identification of *Bacillus velezensis *JR4

Twenty-four endophytic bacteria were isolated from the leaves of healthy cabbage plants collected from different fields in Mansoura city (latitude 31° 3′ 0″ N, longitude 31° 23′ 0″ E, temperature 24–30 °C, loam soil), located in the north of Egypt. The leaves were surface-sterilized with 75% ethanol and 2% sodium hypochlorite for 1 min each, rinsed in sterile distilled water, and dried on sterile filter paper. Leaves were then thoroughly ground, and 100 µL of the resulting fluid was collected after gradient dilution and spread onto nutrient agar (NA) plates. The plates were incubated at 28 °C for 2 to 4 days, after which individual bacterial colonies were isolated and purified.

Molecular identification was performed by extracting genomic DNA from strain JR4 using a bacterial genomic DNA isolation kit (SolarBio, China). JR4 exhibited the highest inhibition rate against the pathogen among all isolated strains in a dual culture test. Strain JR4 was identified through 16 S rRNA analysis using primers 27 F (5′ AGAGTTTGATCMTGGCTCAG 3′) and 1492R (5′ TACGGYTACCTTGTTACGACTT 3′). PCR tubes contained 40 µL of reaction mixture comprising 20 µL of Master Mix, 1 µL DNA template, 0.5 µL of each primer, and 18 µL dH_2_O, and were run in a GeneAmp PCR System 9700 (Applied Biosystems, USA). Amplification involved an initial denaturation at 94 °C for 4 min, followed by 30 cycles of denaturation (94 °C, 30 s), annealing (55 °C, 1 min), and extension (72 °C, 1 min), with a final extension at 72 °C for 10 min. PCR products were separated on a 1% agarose gel. Sequencing was performed by Macrogen Company (Seoul, South Korea). A neighbor-joining method with bootstrap analysis (1000 replicates) was used to construct phylogenetic trees based on 16 S rRNA sequences, incorporating homologous strain sequences from GenBank, using MEGA11 software.

### Antagonism assay

A 5 mm fungal disk of *S. sclerotiorum* was placed in the center of PDA plates, with *B. velezensis* JR4 streaked 2 cm away on either side of the pathogen. Plates with only *S. sclerotiorum* served as controls. All plates were incubated at 26 °C for 5 days, and the experiment was repeated six times. The inhibition rate (IR %) was calculated using the formula: $\mathrm{IR}\% = \left(\mathrm{C}-\mathrm{D}\right)/\mathrm{C}\times 100$, where C and D are the diameters of the pathogen cultures in the control and dual cultures, respectively.

### Detection of antagonist-related lytic enzymes

The ability of *B. velezensis* JR4 to produce protease was tested using a skimmed milk agar plate assay. A 5 µL sample of a 24 h bacterial culture was placed at the center of the plate, which was then incubated at 28 °C for three days. A clear zone around the bacterial colonies, showing hydrolysis, indicated a positive protease activity [27]. Cellulase activity of *B. velezensis* JR4 was evaluated on a carboxymethyl cellulose (CMC) solid medium. A 10 µL bacterial suspension (24 h old) was transferred to the center of the plate. After incubating for 2 days at 28 °C, 0.1% Congo red dye was added for 15 min, followed by rinsing with 1 M NaCl decolorization solution. The appearance of a clear halo around the colonies indicated cellulase production [27]. Chitinase was tested on colloidal chitin medium amended with mineral salts (magnesium sulfate, ammonium sulfate, monopotassium phosphate, citric acid monohydrate), with the pH adjusted to 4.7. A small amount of *B. velezensis* JR4 suspension was placed in the center of the plate, and all plates were incubated at 28 °C for 3 days. Chitinase activity was indicated by a purple zone forming around the bacterial colonies [28]. All experiments were performed in triplicate.

### Biocontrol of *Bacillus velezensis* JR4 against Sclerotinia rot on carrot

The effect of *B. velezensis* JR4 bacterial culture suspension on carrots inoculated with *S. sclerotiorum* was tested following Elsherbiny and Taher [29]. Fresh, uniform-sized carrots at peak commercial maturity, free from physical damage or visible signs of infection, were obtained from local markets in Mansoura City, Egypt. Sterilized carrots (*Daucus carota* cv. Chantenay) were dipped for 10 min in different concentrations of the bacterial suspension (1 × 10^5^, 1 × 10^6^, 1 × 10^7^, and 1 × 10^8^ CFU mL^− 1^) or sterile distilled water as a control. After drying, the carrots were wounded with a sterile needle, and each wound was inoculated with a mycelial disk of *S. sclerotiorum*. All treatments were maintained in plastic boxes at 21 °C with 95% humidity. Each treatment contained three replicates with six carrots per replicate, and the experiment was conducted twice. Disease incidence and lesion length were recorded every 24 h for 5 days.

### Measurement of total phenolics and flavonoids

Total phenolics and flavonoids were measured according to the method of Zheng et al. [30]. For total phenolic content, carrot samples (2 g) treated with *B. velezensis* JR4 culture suspension at 1 × 10^8^ CFU mL^− 1^ or sterile distilled water as a control were ground in 5 mL of 95% ethanol and incubated in the dark at 4 °C for 24 h. After centrifugation, the supernatant (1 mL) was mixed with 0.5 mL of 50% Folin-Ciocalteu reagent, 1 mL of 95% ethanol, and 5 mL of distilled water, then incubated at room temperature for 5 min. Next, 1 mL of Na_2_CO_3_ solution (5%) was added, and the mixture was kept at room temperature for one hour. Absorbance was recorded at 685 nm using gallic acid as the standard, and results were expressed as milligrams per gram of carrot tissue.

For total flavonoids, carrot samples (2 g) were ground in 2 mL of 70% ethanol and centrifuged at 12,000 × *g* for 10 min. Then, 1 mL of the supernatant was combined with 0.3 mL of 5% NaNO_2_ and 0.3 mL of 10% AlCl_3_, left at room temperature for 5 min, and then 2 mL of 1 M NaOH was added. Absorbance was measured at 510 nm, and the results were expressed as milligrams of catechin per gram of carrot tissue. All measurements were performed in triplicate.

### Determination of cell membrane permeability and malondialdehyde (MDA) content

Cell membrane permeability, measured as relative electrical conductivity, was assessed following the method of Yang et al. [31]. Carrot disks (0.1 cm thick, 1 cm diameter) treated with a culture suspension of *B. velezensis* JR4 (1 × 10^8^ CFU mL^− 1^) or sterile distilled water as a control were rinsed with deionized water. The samples were shaken in deionized water for 30 min at 25 °C, and conductivity was measured using an electrical conductivity meter (Jenco 3173, Jenco Technologies, USA). The solution was then boiled for 15 min, rapidly cooled, and the final conductivity was recorded. Relative conductivity (%) was calculated as: conductivity / final conductivity × 100. The experiment was repeated twice with three independent replicates for each treatment.

MDA levels were determined using the method from Zhang et al. [32]. Carrot samples (1 g) were ground in phosphate buffer (pH 7.8) and centrifuged at 10,000 × *g* for 10 min. Two milliliters of the supernatant were mixed with 3 mL of 0.2% thiobarbituric acid (TBA) in 20% trichloroacetic acid (TCA), heated at 100 °C for 10 min, quickly cooled on ice, and centrifuged again at 10,000 × *g* for 10 min. The absorbance of the supernatant was measured at 532 nm, and MDA content was expressed as µmol per gram of fresh weight. All experiments were performed in triplicate.

### Determination of superoxide anion (O_2_^−^), and hydrogen peroxide (H_2_O_2_)

The superoxide anion production rate was determined following the method of Fagundes-Nacarath et al. [33]. One gram of the carrot sample treated with *B. velezensis* JR4 culture suspension at 1 × 10^8^ CFU mL^− 1^ or from the control group was ground and mixed with 2 mL of 100 mM sodium phosphate buffer (pH 7.2) containing 1 mM sodium diethyldithiocarbamate (SDD), then centrifuged at 10,000 × *g* for 10 min at 4 °C. The resulting supernatant was blended with 2 mL of 100 mM sodium phosphate buffer (pH 7.2), 1 mM SDD, and 0.25 mM nitroblue tetrazolium (NBT). Absorbance was measured at 540 nm, and O_2_^−^ concentration was reported as µmol per gram of fresh weight.

Hydrogen peroxide levels were measured following the method of Kumar et al. [34]. One gram of carrot samples was mixed with 0.1% trichloroacetic acid (TCA) and centrifuged at 12,000 × *g* for 15 min at 4 °C. The resulting supernatant (0.5 mL) was mixed with 1 M potassium phosphate buffer (pH 7.0) and 1 mL of 1 M potassium iodide (KI) solution, then incubated for 5 min. Absorbance was read at 390 nm, and hydrogen peroxide content was determined using a standard calibration curve prepared with various H_2_O_2_ concentrations. Results were expressed as µmol per gram of fresh weight. All experiments were performed in triplicate.

### Assignment of defense-related enzyme activity

Carrot samples treated with *B. velezensis* JR4 at 1 × 10^8^ CFU mL^− 1^, along with control samples, were collected after 1, 2, 3, and 4 days to measure the activity of peroxidase (POD), polyphenol oxidase (PPO), and phenylalanine ammonia-lyase (PAL). All measurements were performed in triplicate.

The guaiacol method was used to measure the activity of POD and PPO [35]. Briefly, carrot tissue (2 g) was ground in 100 mM sodium phosphate buffer (pH 6.4) with 1% polyvinylpolypyrolidone (PVPP), then centrifuged at 12,000 × *g* for 30 min at 4 °C. The supernatant was used for enzyme assays. Protein content was measured using bovine serum albumin (BSA) as the standard, following the method described by Lowry et al. [36]. POD and PPO activity were expressed in units (U), where one unit is defined as the change in absorbance at 460 nm and 398 nm per mg of protein per minute, respectively. PAL activity was measured using the method described by Sun et al. [37]. Carrot tissue (1 g) was ground in a solution containing 200 mM boric acid buffer (pH 8.8), 50 mM β-mercaptoethanol, 1 mM ethylenediaminetetraacetic acid (EDTA), and 10% PVPP. Activity was defined as the change in absorbance at 290 nm per milligram of protein per minute and expressed in units (U).

### Effect on the expression of defense-related genes

Total RNA was extracted from control and treated carrot samples exposed to a culture suspension of *B. velezensis* JR4 at 1 × 10^8^ CFU mL^− 1^ after 1, 2, 3, and 4 days, using TRIzol reagent (Invitrogen) according to the instructions. RNA purity and quality were assessed with a Nanodrop spectrophotometer (Thermo Scientific, USA). RNA integrity was assessed using gel electrophoresis on a 1% agarose gel. Genomic DNA contamination was removed with DNase I treatment (Thermo Scientific, USA). First-strand cDNA was synthesized using the RevertAid First Strand cDNA Synthesis Kit (Thermo Scientific, USA). qRT-PCR was carried out using PowerUp SYBR Green Master Mix (Applied Biosystems, USA) on a QuantStudio 3 real-time PCR system (Applied Biosystems, USA). qRT-PCR was performed in a final volume of 20 µL, containing 10 µL of SYBR Green, 2 µL of cDNA, 2 µL each of forward and reverse primers, and 4 µL of nuclease-free water. Primer sequences used for qRT-PCR are listed in Table 1. The Actin gene served as an internal reference to normalize target gene expression. Gene expression was assessed using three biological replicates and two technical replicates per reaction, under the following conditions: 95 °C for 2 min, followed by 40 cycles of 95 °C for 15 s, 60 °C for 30 s, and 72 °C for 30 s. Relative expression levels were calculated using the 2^−ΔΔCt^ method [37].

**Table 1:** Nucleotide sequences of primers used for qRT-PCR analysis

Gene	Forward primer 5′-3′	Reverse primer 5′-3′
POD	CCCGCAAAATGGTGATGGAA	GTCAGTCCAGCCAATCCTCT
PPO	GCGGAGATACCAAGCAGCCAAA	GCGTCTTCCAAACCGTCCACAT
PAL	AGGGCAAACCCGAATTTACTGAC	CATAAGAGCTTCCATCCAAGATG
Actin	ACCGTATGAGCAAGGAAATCACTG	TCGTCGTACTCACCCTTTGAAATC

### Cell viability and cytotoxicity assay

*B. velezensis* JR4 was cultured in nutrient broth (NB) medium and incubated at 28 °C for 48 h with shaking. The culture was then centrifuged at 10,000 × *g* for 15 min at 10 °C, and the supernatant was sterilized through a 0.22 μm pore size filter to obtain a cell-free culture filtrate. For the cytotoxicity test, Vero cells (ATCC: CCL-81), derived from the kidney epithelial cells of an African green monkey, and WI-38 cells (ATCC: CCL-75), which are diploid human fibroblasts from the lung tissue of a three-month-old female fetus, were used. The cytotoxicity of *B. velezensis* JR4 was assessed using the MTT method (3-[4,5-dimethylthiazol-2-yl]-2,5-diphenyl tetrazolium bromide) [38]. The cells under examination were seeded into a 96-well plate and treated with varying concentrations of cell-free culture filtrate of *B. velezensis* JR4. The plates were incubated for 24 h at 37 °C, then the monolayer was washed twice and incubated again in RPMI medium with 2% serum for 48 h. Each dilution was tested in separate wells, with control wells containing only RPMI. Afterward, the medium was removed, and an MTT solution (5 mg mL^− 1^ in PBS) was added. The plates were incubated at 37 °C for 4 h. MTT crystals were mixed with 200 µL of 10% DMSO in each well, and all samples were shaken at 150 rpm for 30 min in the dark to fully dissolve the formazan in the solvent. Absorbance was recorded at 570 nm, and changes in the morphology of the cells were observed using a phase-contrast microscope. Cell viability was calculated using the formula from Ibrahim et al. [39].

### Statistical analysis

The data are presented as mean values ± standard error (SE). Statistical analysis was conducted using the Statistical Analysis System (SAS Institute Inc., NC, USA). One-way ANOVA was performed on all variables, and Tukey’s HSD test was used to compare significant differences between the treatment and control groups at the same time point. Significant differences were marked with one asterisk (*) for *P* < 0.05, and highly significant differences with two asterisks (**) for *P* < 0.01. Furthermore, different letters were used to indicate significant differences (*P* < 0.05).

## Results

### Molecular identification of *Bacillus velezensis* JR4

**Fig. 1:**
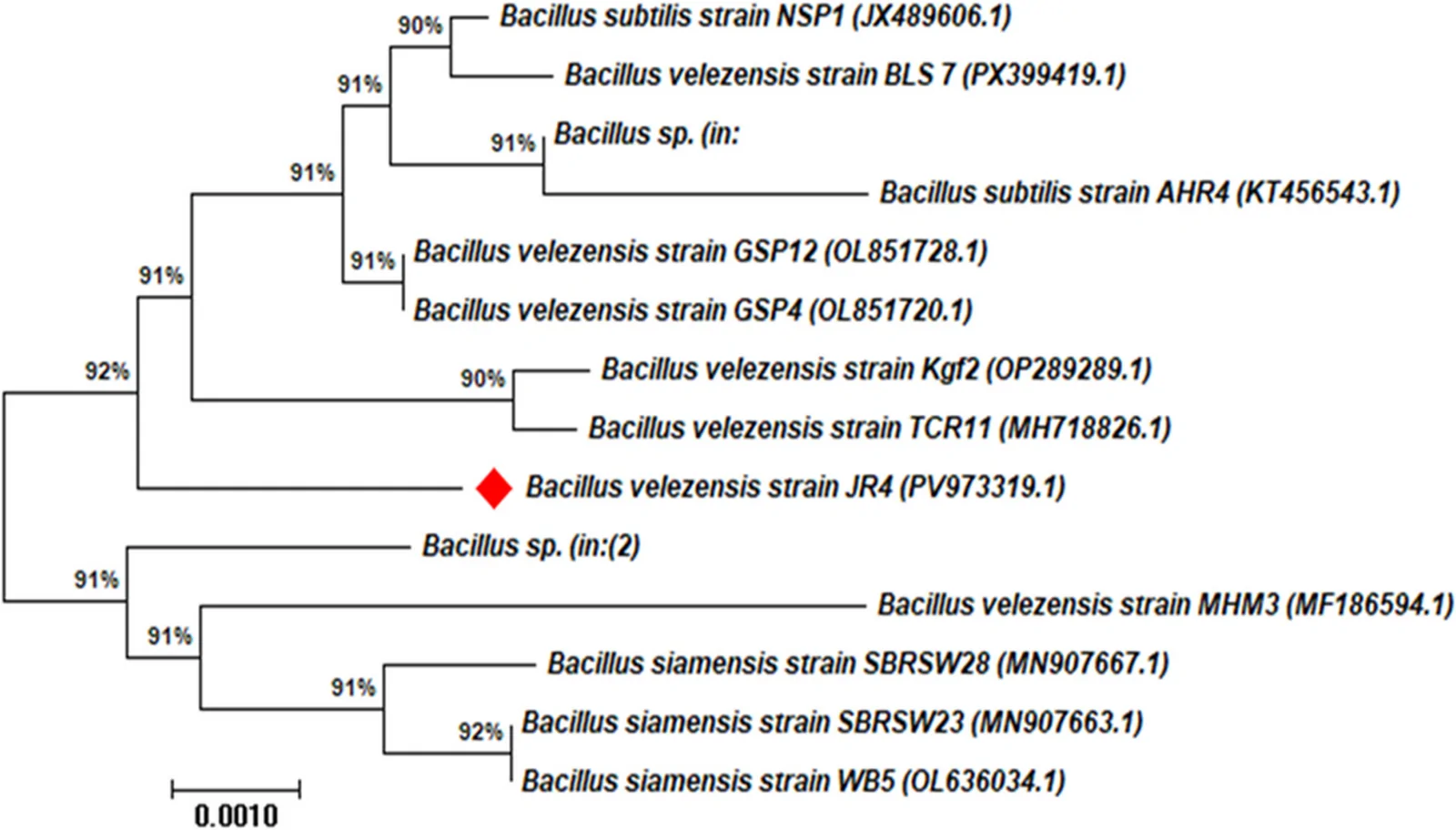
Phylogenetic tree based on 16 S rRNA gene sequence of *Bacillus velezensis* JR4 (GenBank Accession No. PV973319.1) using the neighbor-joining method in MEGA11. Bootstrap value of 1000 replicates

The phylogenetic tree based on the 16 S rRNA sequences of JR4 (GenBank accession number: PV973319.1) and other *Bacillus* strains revealed that JR4 is closely related to *B. velezensis* strains (Fig. 1). Subsequent phylogenetic tree analysis and sequencing showed that strain JR4 shares 99.55% similarity with *B. velezensis* strains GSP4, TCR11, and GSP12 with accession numbers OL851720.1, MH718826.1, and OL851728.1.

### Antagonistic activity of *Bacillus velezensis* JR4 on *Sclerotinia sclerotiorum*

**Fig. 2:**
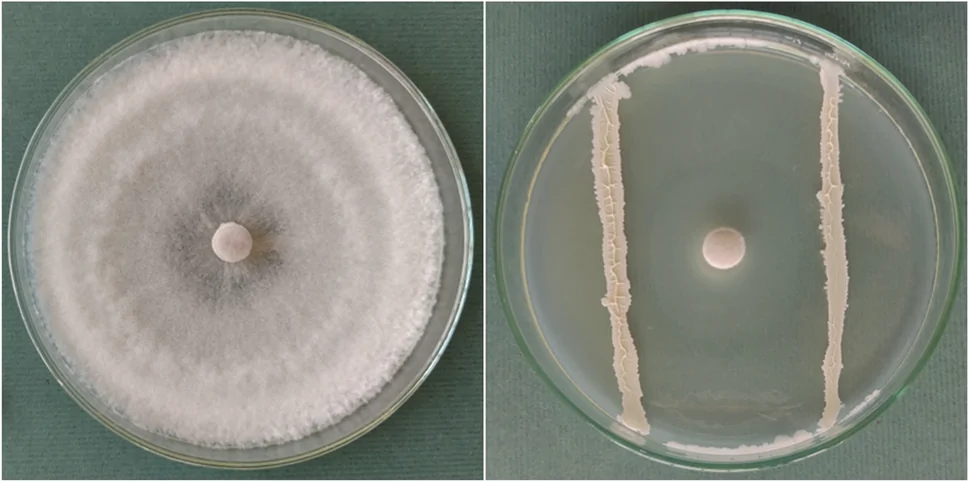
The dual culture of *Sclerotinia sclerotiorum* and *Bacillus velezensis* JR4 on PDA plates. Left: *S. sclerotiorum* (control); Right: *S. sclerotiorum* in the presence of *B. velezensis* JR4

The percentage of fungal growth inhibition by *B. velezensis* JR4 was determined by measuring the reduction in *S. sclerotiorum* growth in dual culture plates compared to control plates. The results showed that the endophytic bacterium *B. velezensis* strain JR4 caused an inhibition rate up to 91.1% against *S. sclerotiorum* (Fig. 2).

### Detection of extracellular enzymes production

**Fig. 3:**
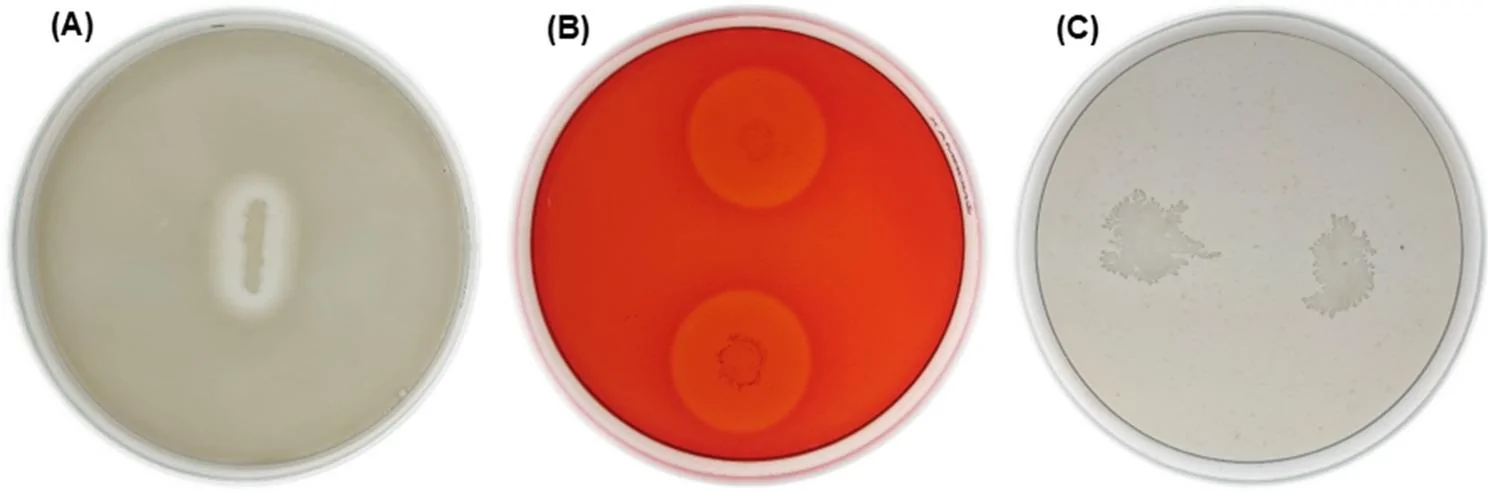
Production of extracellular enzymes by *Bacillus velezensis* JR4 in vitro. **A** Protease activity was detected using a skimmed milk agar plate medium. **B** Cellulase activity was detected on a carboxymethyl cellulose medium. **C** Chitinase activity was detected on a colloidal chitin medium amended with mineral salts

The ability of *B. velezensis* JR4 to produce extracellular enzymes was examined to study the antagonistic mechanisms. The results showed that the isolate JR4 was highly capable of producing protease, cellulase, and chitinase, with the development of clear zones formed around colonies of *B. velezensis* JR4 on the skimmed milk agar plate medium (Fig. 3A), carboxymethyl cellulose medium (Fig. 3B), and colloidal chitin medium amended with mineral salts (Fig. 3C), respectively.

### Disease control efficacy

**Fig. 4:**
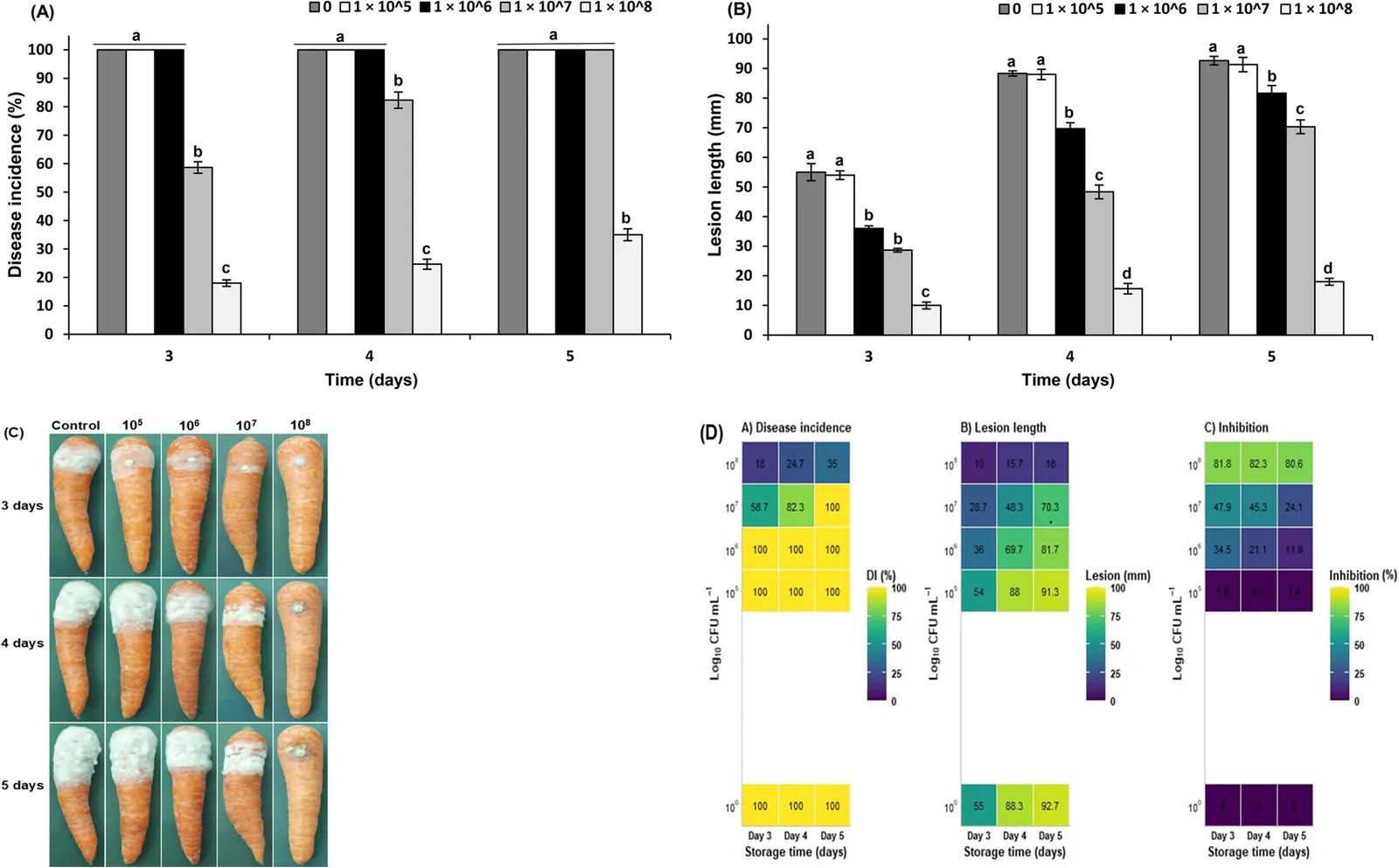
Effect of bacterial suspension of *Bacillus velezensis* JR4 at different concentrations (1 × 105, 1 × 106, 1 × 107, and 1 × 108 CFU mL⁻¹) on (**A**) disease incidence, (**B**) lesion length, and (**C**) disease phenotype of rot in postharvest carrot inoculated with *Sclerotinia sclerotiorum* with (**D**) Z-score heatmap of disease incidence, lesion length, and inhibition rate. Each bar represents the mean ± standard error (SE). Columns with different letters are significantly different at the *P* < 0.05 level according to Tukey’s HSD test

The effects of *B. velezensis* JR4 suspension at different concentrations, 1 × 10^5^, 1 × 10^6^, 1 × 10^7^, and 1 × 10^8^ CFU mL^− 1^, on rot incidence, and lesion length in postharvest carrots inoculated with *S. sclerotiorum* after 3, 4, and 5 days are shown in Fig. 4. The results presented in Fig. 4A showed that the treatment with JR4 at 1 × 10^8^ CFU mL^− 1^ caused a significant reduction (*P* < 0.05) of 82.1, 75.3, and 65.4% in rot incidence on days 3, 4, and 5, respectively, in comparison with the control. In this context, a concentration of 1 × 10^8^ CFU mL^− 1^ significantly reduced lesion length (*P* < 0.05) on carrots by 81.8, 82.3, and 80.6% on days 3, 4, and 5, respectively, compared to the control at all storage times (Fig. 4B, C,D). A concentration of 1 × 10^7^ CFU mL^− 1^ also led to a noticeable decrease in lesion length, particularly after 3 and 4 days of pathogen inoculation, resulting in a 47.9 and 45.3% reduction relative to the control (Fig. 4B, C,D).

### Total phenolics and flavonoids

**Fig. 5:**
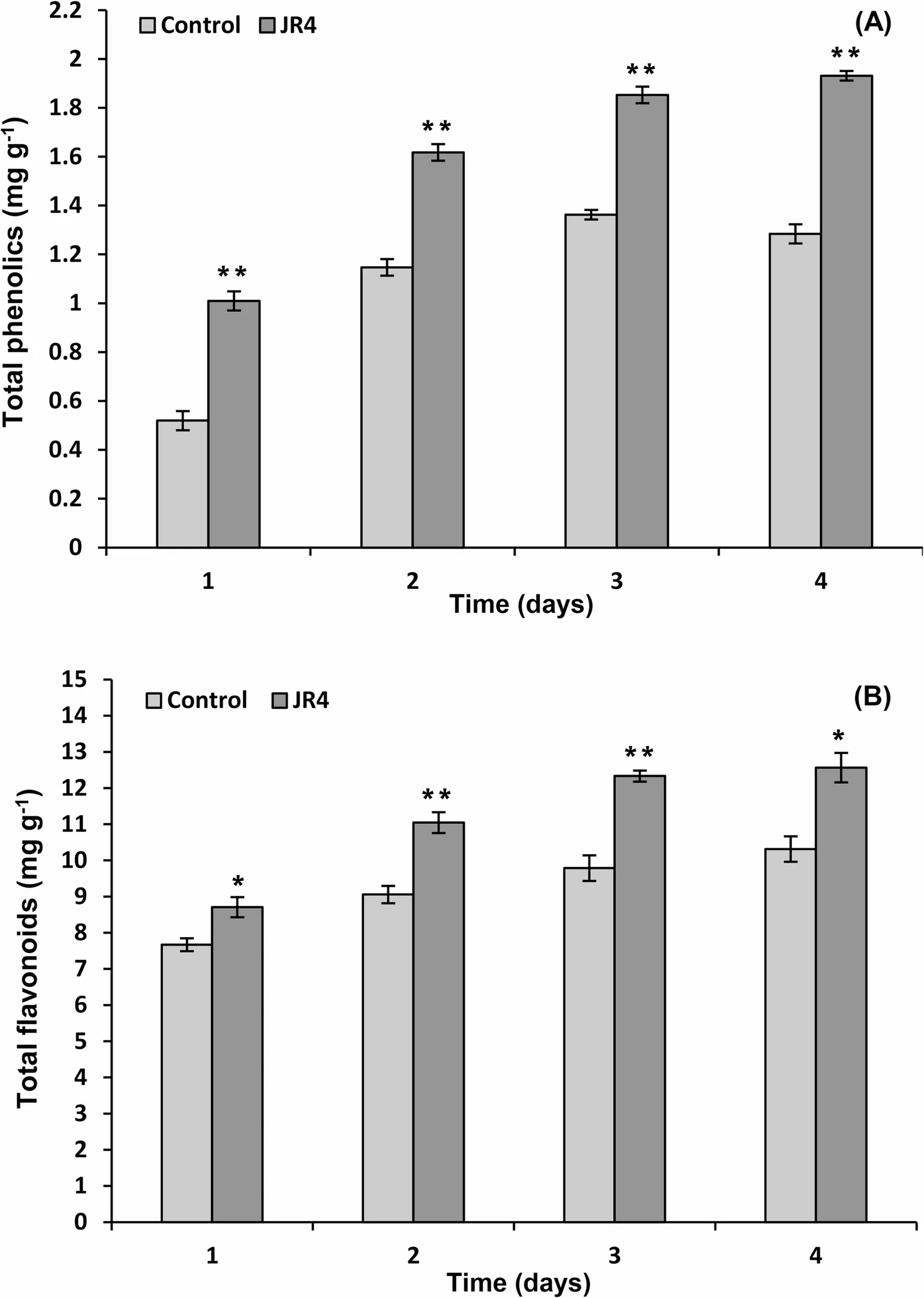
Effect of bacterial suspension of *Bacillus velezensis* JR4 at the concentration of 1 × 10^8^ CFU mL^− 1^ on the total content of phenolics (**A**) and flavonoids (**B**) in postharvest carrot for four days. Each bar represents the mean ± standard error (SE). A single asterisk (*) indicates a significant difference from the control group (*P* < 0.05), while double asterisks (**) indicate a highly significant difference (*P* < 0.01) for the same time point

The levels of total phenolics and flavonoids in postharvest carrots were monitored over 4 consecutive days after treatment with JR4 at 1 × 10^8^ CFU mL^− 1^, as shown in Fig. 5. Treated carrots showed a significant increase in total phenolics compared to the control at all measured times, and phenolics content reached its maximum level on the fourth day (Fig. 5A), which was 1.51 times higher than the control (*P*-value: < 0.001). Compared with the control, total flavonoid contents in treated postharvest carrots significantly increased over 4 days of storage (Fig. 5B), with the highest concentrations of flavonoid found during the last three days of the experiment.

### Cell membrane permeability, malondialdehyde (MDA), superoxide anion (O_2_^−^), and hydrogen peroxide (H_2_O_2_)

**Fig. 6:**
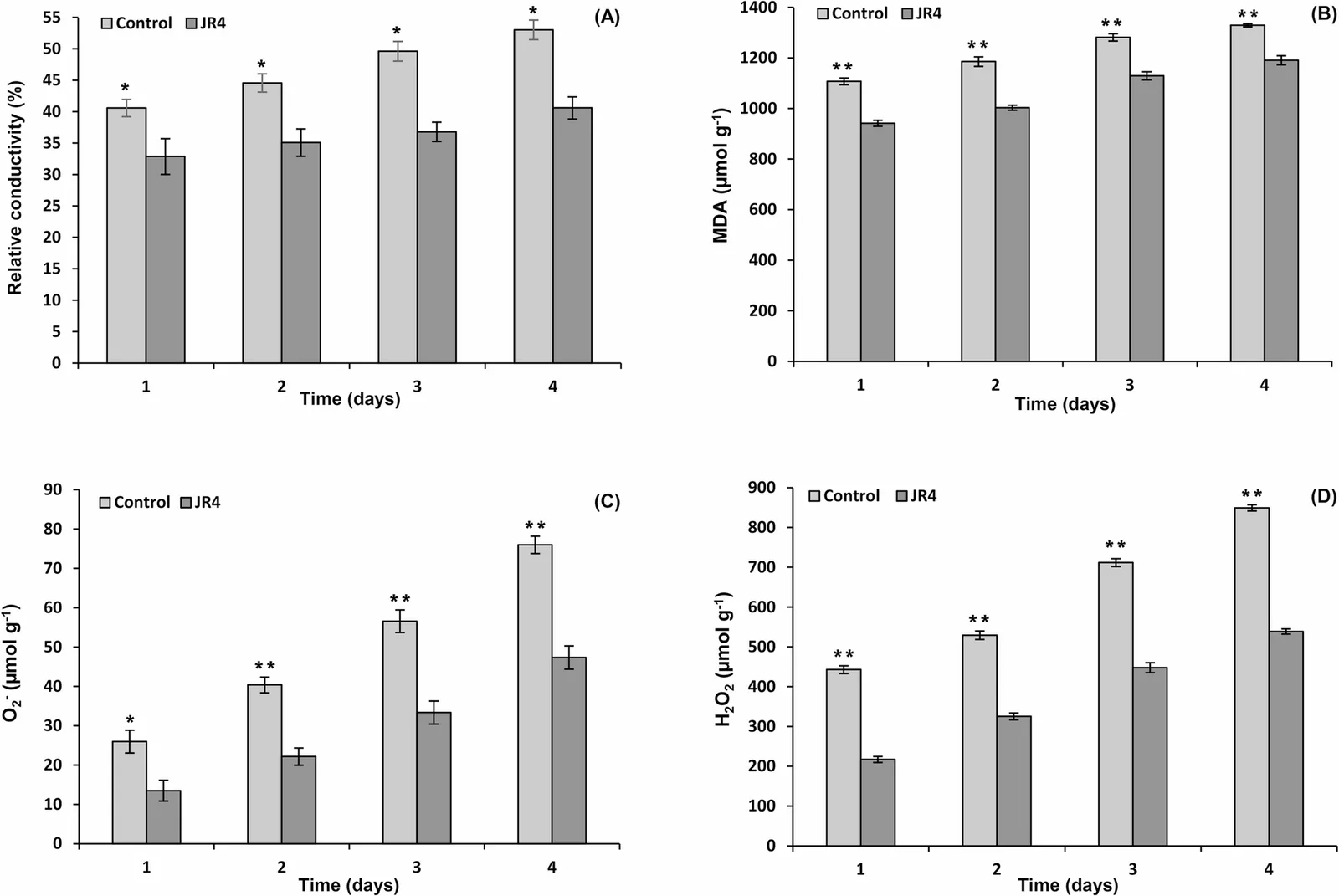
Effect of bacterial suspension of *Bacillus velezensis* JR4 at 1 × 10^8^ CFU mL^− 1^ on the levels of (**A**) cell membrane permeability, (**B**) malondialdehyde (MDA), (**C**) superoxide anion (O_2_^−^), and (**D**) hydrogen peroxide (H_2_O_2_) in postharvest carrot for four days. Results represent the mean ± standard error (SE). A single asterisk (*) indicates a significant difference from the control group (*P* < 0.05), while double asterisks (**) indicate a highly significant difference (*P* < 0.01) for the same time point

In postharvest carrots, both the control group and those treated with *B. velezensis* JR4 suspension (1 × 10^8^ CFU mL^− 1^) showed a gradual increase in relative membrane permeability during the experiment (Fig. 6A). However, the bacterial suspension significantly slowed (*P* < 0.05) the increase in cell membrane permeability compared to the control on all days of the experiment. On day 4 of storage, each group reached its peak relative conductivity. The control group recorded the highest value at 53.1%, while the JR4 treatment group exhibited the lowest at 40.6%. The MDA content in carrots treated with JR4 was lower than that in the untreated carrots (Fig. 6B), with a significant difference (*P* < 0.01) noted between the treated and control groups across all 4 days of the experiment. By day 4 of treatment, the MDA levels in the control and treatment groups were 1329.1 µmol g^− 1^ and 1190.5 µmol g^− 1^, respectively (*P*-value: < 0.002).

JR4 at 1 × 10^8^ CFU mL^− 1^ caused a remarkable decrease in the O_2_^−^ production rate in carrot fruit compared to that of the control group after 1, 2, 3, and 4 days of storage (Fig. 6C). For example, on day 4, the O_2_^−^ level was 47.3 µmol g^− 1^ in the treated carrots, compared to 75.9 µmol g^− 1^ in the untreated group (*P*-value: < 0.001). Meanwhile, the H_2_O_2_ levels in carrot fruit treated with JR4 were significantly reduced (*P* < 0.01) during the storage period compared to the control (Fig. 6D). After 4 days of treatment, the H_2_O_2_ content was 849.2 µmol g^− 1^ in the control group, while it was only 538.7 µmol g^− 1^ in the JR4 group.

### Defense-related enzyme activities

**Fig. 7:**
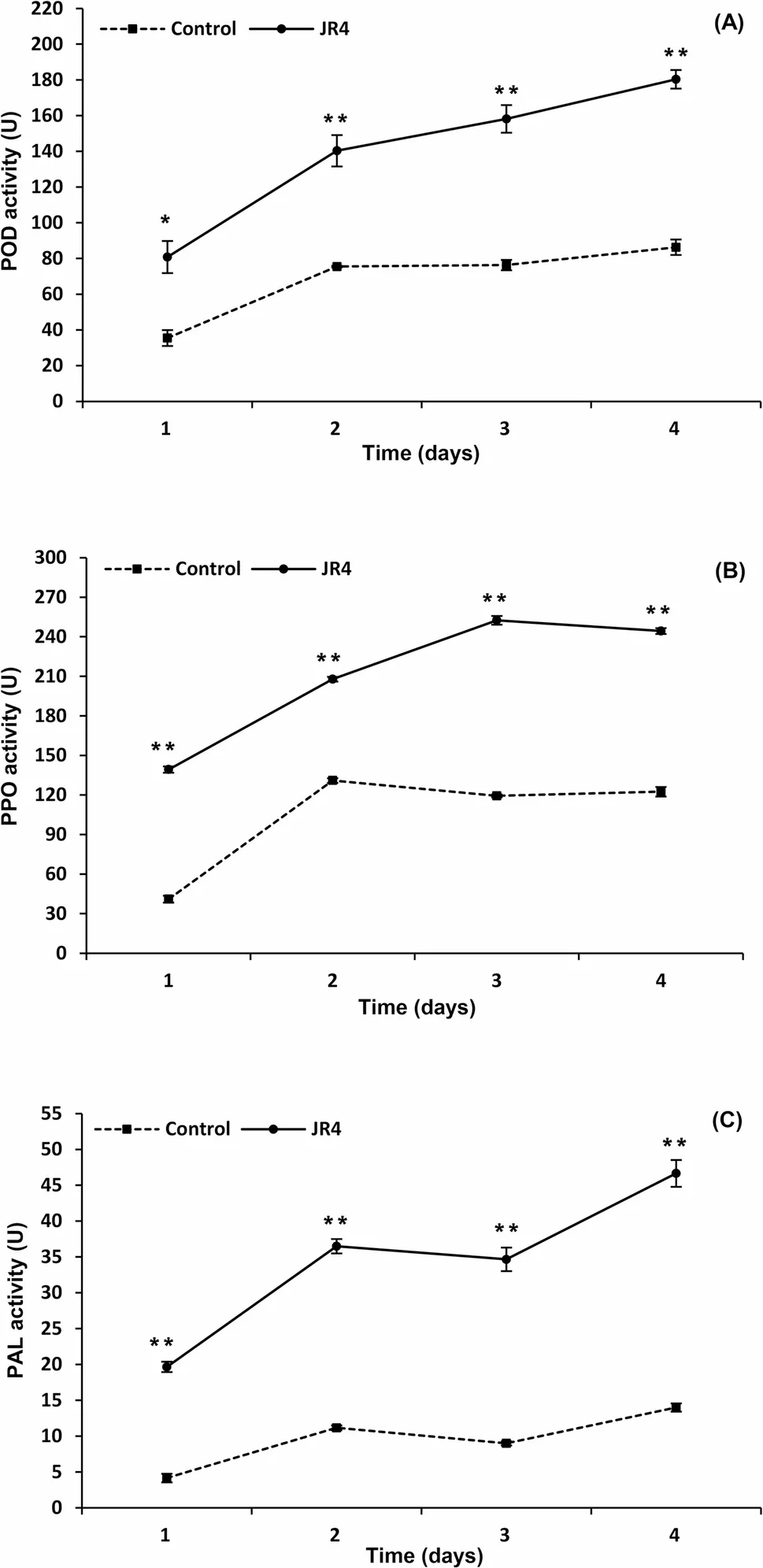
Effect of bacterial suspension of *Bacillus velezensis* JR4 at the concentration of 1 × 10^8^ CFU mL^− 1^ on the activity of (**A**) peroxidase (POD), (**B**) polyphenoloxidase (PPO), and (**C**) phenylalanine ammonia-lyase (PAL) in postharvest carrot for four days. Results represent the mean ± standard error (SE). A single asterisk (*) indicates a significant difference from the control group (*P* < 0.05), while double asterisks (**) indicate a highly significant difference (*P* < 0.01) for the same time point

Treatment with JR4 at 1 × 10^8^ CFU mL^− 1^ significantly increased the POD activity in postharvest carrots in comparison with the untreated group during the entire experimental period (Fig. 7A), with activity peaking on days three and four at roughly 2.1 times higher than the control. PPO activity was significantly higher (*P* < 0.01) in the group treated with JR4 compared to the control group during storage (Fig. 7B). In treated postharvest carrots, PPO activity increased sharply during the first three days, then began to decline slightly on the last day of the experiment. The PAL activity in carrots treated with JR4 was significantly higher (*P* < 0.01) than that of the control during the whole storage period (Fig. 7C). However, PAL activity declined slightly on the third day but increased again by the final day of the experiment. The maximum PAL values were 3.8 and 3.3 times greater than the control on the third and fourth days, respectively.

### Expression analysis of defense-related genes

**Fig. 8:**
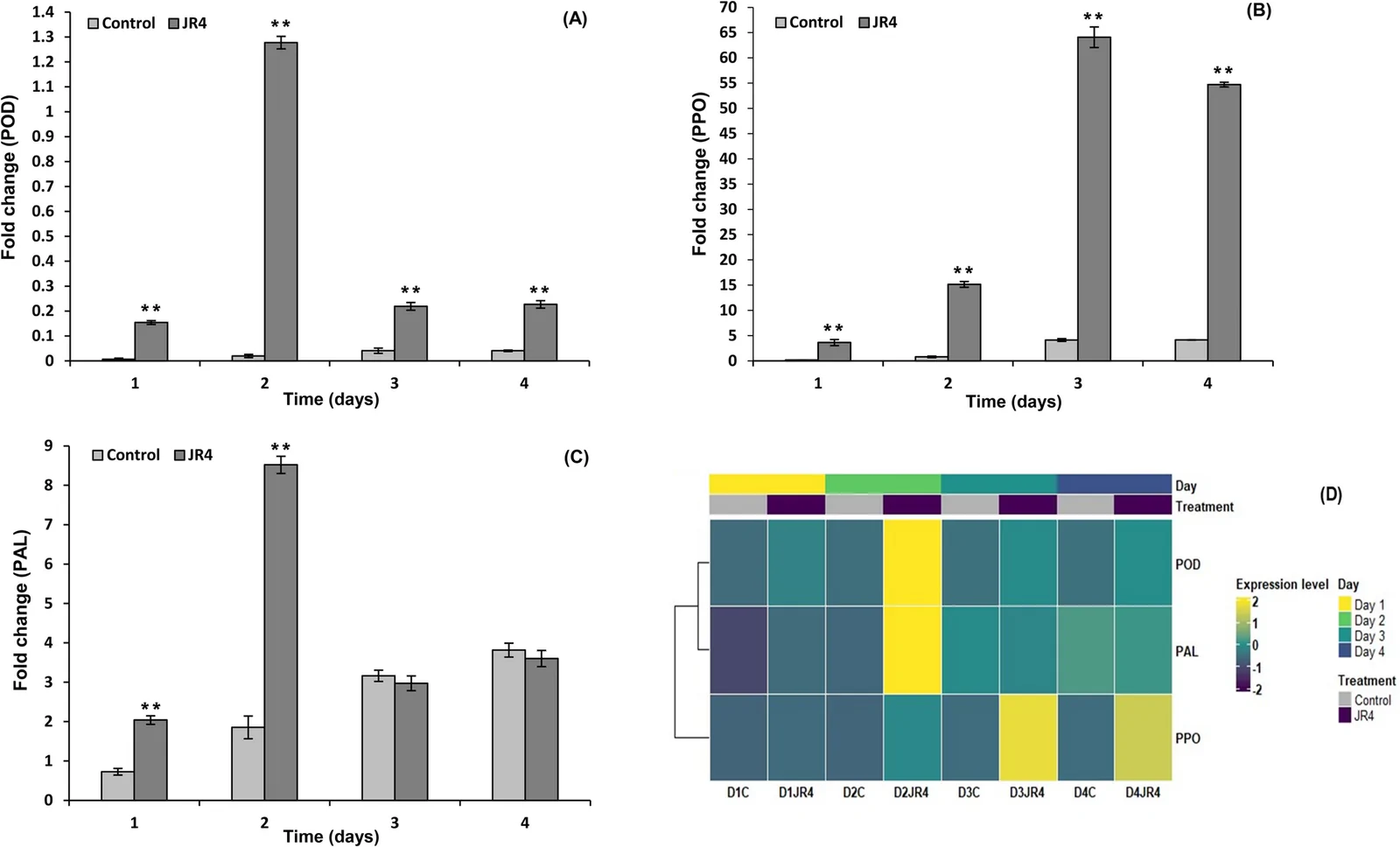
Effect of bacterial suspension of *Bacillus velezensis* JR4 at the concentration of 1 × 10^8^ CFU mL^− 1^ on the gene expressions of (**A**) peroxidase (POD), (**B**) polyphenoloxidase (PPO), and (**C**) phenylalanine ammonia-lyase (PAL) in postharvest carrot for four days, with (**D**) Z-score heatmap of gene expression profiles. qRT-PCR was performed using actin as the reference gene. The results represent the mean of three independent replicates. Results represent the mean ± standard error (SE). A single asterisk (*) indicates a significant difference from the control group (*P* < 0.05), while double asterisks (**) indicate a highly significant difference (*P* < 0.01) for the same time point

Real-time quantitative PCR was used to measure the expression of three defense-related genes in postharvest carrots treated with a JR4 bacterial suspension at 1 × 10^8^ CFU mL^− 1^ over 1, 2, 3, and 4 days. Compared to the control on day 1, all three genes showed a significant increase (*P* < 0.01) in expression levels (Fig. 8A, B,C). At day 2, the expressions of POD and PAL in treated carrots were 66.8 and 4.6 times higher, respectively, than in the control. In particular, PPO expression in the treated carrots was 15.5 and 13.2 times higher than in the control on days 3 and 4, respectively. Meanwhile, there was no significant difference (*P* < 0.05) in PAL levels between the two groups during those days.

The Z-score heatmap of gene expression profiles revealed a clear difference in the regulation of three defense-related genes (POD, PPO, and PAL) between JR4-treated samples and controls over storage time (Fig. 8D). JR4 treatment consistently induced the expression of these genes, as indicated by the positive Z-scores, while the control group showed lower expression levels. The clustering pattern further supports time-dependent, treatment-specific transcriptional changes, pointing to a coordinated gene expression network.

### Multivariate analysis

**Fig. 9:**
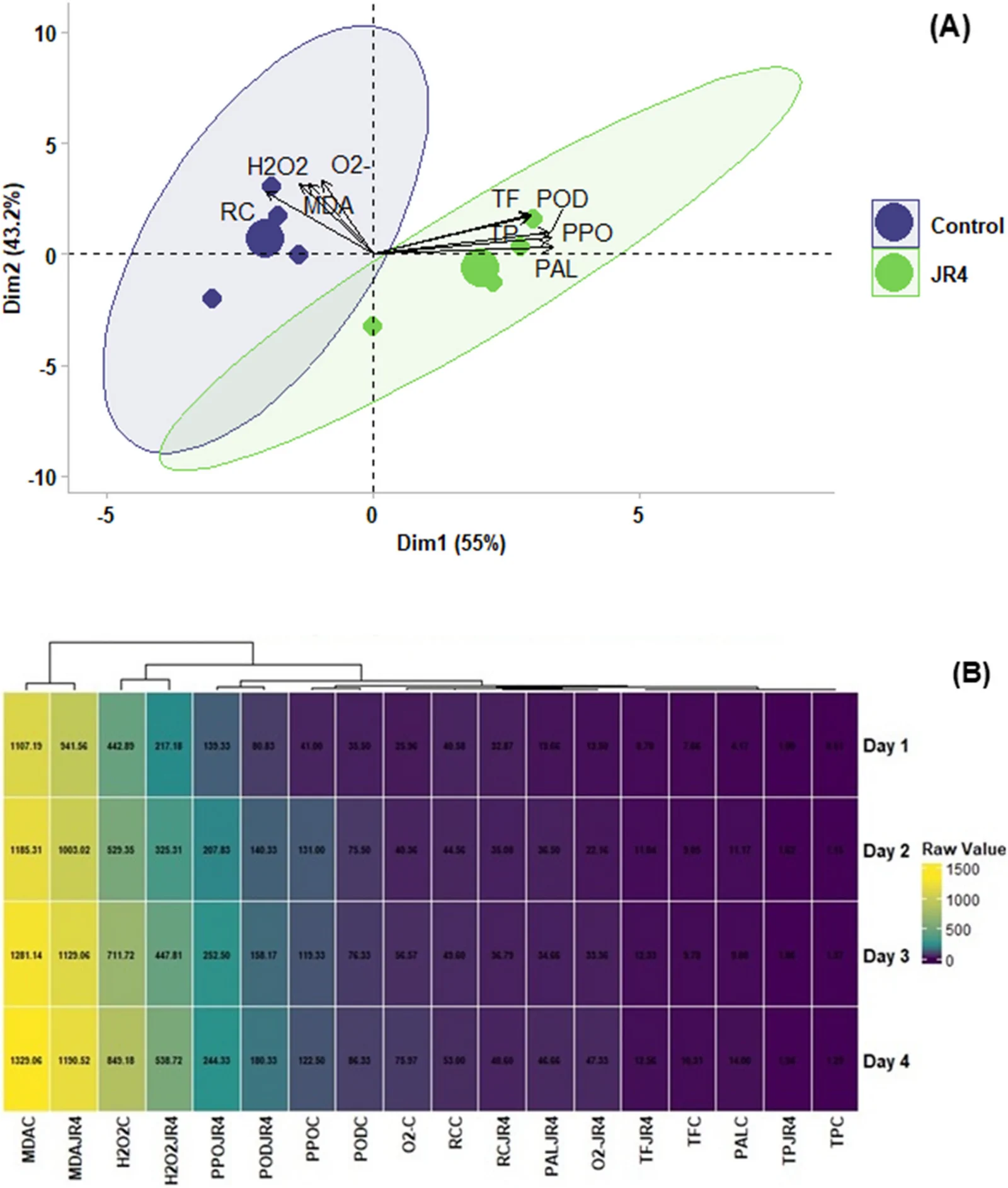
Multivariate analysis of physiological and biochemical responses under *Bacillus velezensis* JR4 treatment and control. **A** Principal component analysis (PCA) based on all measured physiological and biochemical parameters. The first two principal components, PC1 (55%) and PC2 (43.2%), together explained 98.2% of the total variance. Arrows indicate the direction and magnitude of each variable’s contribution to the principal components. **B** Hierarchical heatmap showing the raw physiological and biochemical measurements across treatments and sampling times. The heatmap uses the Viridis color scale, where purple indicates lower values, green indicates intermediate values, and yellow indicates higher values. Hierarchical clustering groups variables with similar response patterns

The PCA biplot showed a clear split between JR4-treated and control samples based on combined biochemical responses (Fig. 9A). The first two principal components explained most of the variation among antioxidant enzymes (PPO, POD, and PAL), phenolic compounds (TPC and TFC), and oxidative stress markers (MDA, O_2_^−^, and H_2_O_2_), highlighting strong relationships among these factors. JR4-treated samples cluster distinctly from the controls, pointing to a shift toward higher antioxidant capacity and lower oxidative stress. The loadings also revealed that defense-related metabolites are associated with the JR4 cluster, while oxidative damage markers align with the control group.

The heatmap showed that JR4 treatment triggered a coordinated activation in the fruit’s defense metabolism, evidenced by consistent increases in PPO, POD, PAL, TPC, and TFC, as well as a significant decrease in oxidative stress indicators such as MDA, O_2_^−^, and H_2_O_2_, when compared to the control group. The clustering pattern distinctly separated defense responses from signs of oxidative damage, highlighting a clear, time-based physiological reprogramming (Fig. 9B).

### Cell viability and cytotoxicity test

**Fig. 10:**
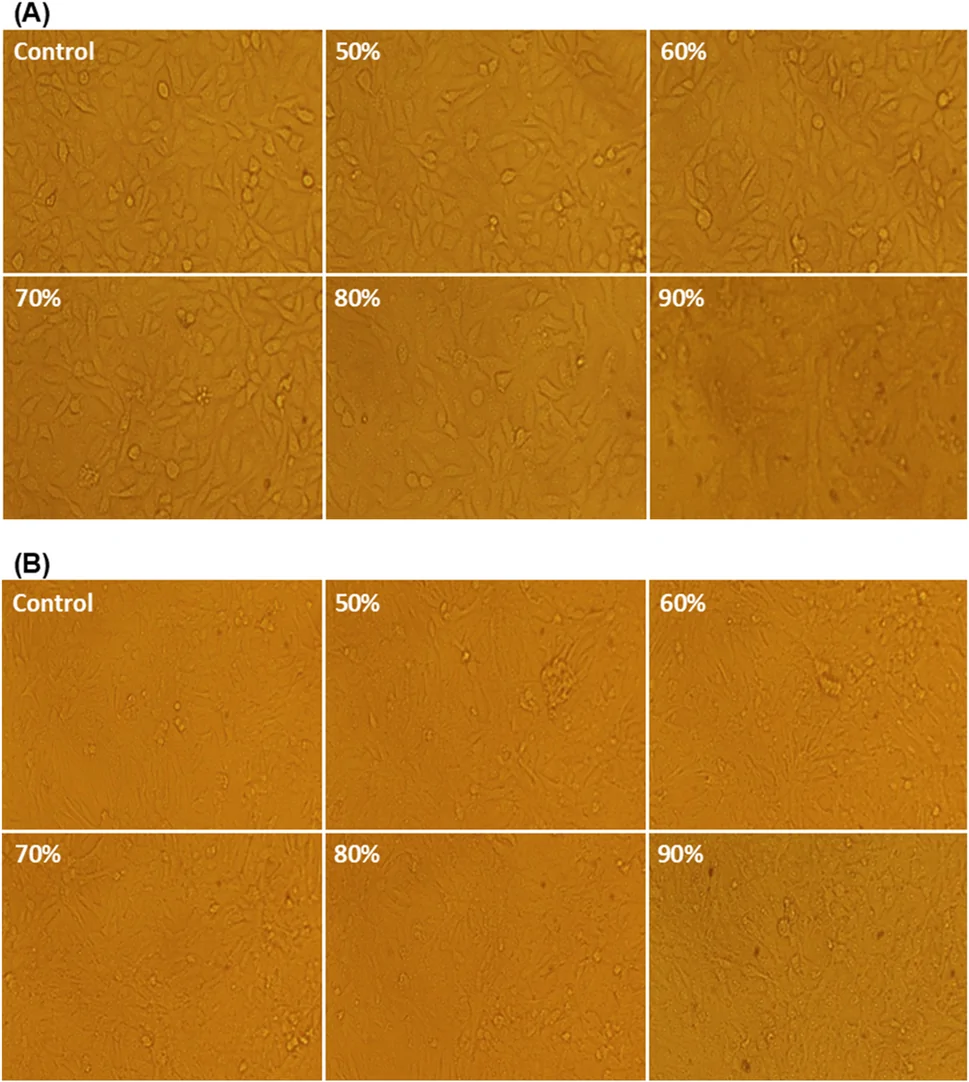
Effect of cell-free culture filtrate of *Bacillus velezensis* JR4 at different concentrations on (**A**) WI-38 cells (ATCC: CCL-75), diploid human fibroblasts derived from the lung tissue of a three-month-old female fetus, and (**B**) Vero cells (ATCC: CCL-81), kidney epithelial cells from an African green monkey

The results showed no significant differences (*P* < 0.05) in cell viability for concentrations between 50% and 80% of the cell-free culture filtrate of *B. velezensis* JR4 in both WI-38 and Vero cells (Table 2). WI-38 cell viability ranged from 99.47% to 100%, while Vero cell viability was between 98.58% and 100%. However, at the highest concentration of 90% from culture filtrate, a significant decrease (*P* < 0.05) in cell viability was observed, with WI-38 and Vero cells showing viability of 82.31% and 89.46%, respectively. The cytotoxicity tests showed that the culture filtrate of JR4 caused no or very minimal toxicity at all concentrations (Fig. 10A, B). As shown in Table 2, toxicity ranged from 0 to 0.52% for WI-38 cells and 0 to 0.41% for Vero cells. At the highest bacterial filtrate concentration of 90%, cytotoxicity reached 17.68% for WI-38 cells and 10.53% for Vero cells.

**Table 2:** Cell viability and cytotoxicity levels of cell-free culture filtrate of *Bacillus velezensis* JR4 on WI-38 cells and Vero cells at different concentrations

Concentration (%)	WI-38 cells		Vero cells	
	Viability (%)	Cytotoxicity (%)	Viability (%)	Cytotoxicity (%)
Control	100 ± 0.0 a	0 ± 0.0 c	100 ± 0.0 a	0 ± 0.0 c
50	100 ± 0.0 a	0 ± 0.0 c	100 ± 0.0 a	0 ± 0.0 c
60	100 ± 0.0 a	0 ± 0.0 c	100 ± 0.0 a	0 ± 0.0 c
70	99.95 ± 0.2 a	0.04 ± 0.3 b	99.95 ± 0.2 a	0.04 ± 0.2 b
80	99.47 ± 0.3 a	0.52 ± 0.3 b	99.58 ± 0.2 a	0.41 ± 0.3 b
90	82.31 ± 0.6 b	17.68 ± 0.4 a	89.46 ± 0.5 b	10.53 ± 0.3 a

Results represent mean ± standard error (SE). Different letters in the same column are significantly different according to Tukey’s HSD test at *P* < 0.05

## Discussion

The results of this study revealed that *B. velezensis* JR4 exhibited a strong antagonistic effect on *S. sclerotiorum*, with 91.1% inhibition. The ability of *B. velezensis* to function as a biocontrol agent is associated with its capacity to produce bioactive secondary metabolites [40]. Research has shown that *B. velezensis* produces bioactive compounds, including polyketides (e.g., difficidin, bacillaene, oxidifficidin, and macrolactin), peptides (e.g., amylocyclicin, bacilysin, and plantazolicin), and cyclic lipopeptides (e.g., bacillomycin D, fengycin, and surfactin), which are released into the surrounding environment [21]. Similarly, biochemical characterization revealed that *B. velezensis* JR4 produces hydrolytic enzymes, including protease, cellulase, and chitinase. This production might also contribute to its antagonistic effect. Proteases and chitinases degrade the cell wall components of fungal pathogens, thereby weakening cell integrity, and can also act as MAMPs (microbe-associated molecular patterns), triggering various mechanisms associated with plant defense [41, 42]. Furthermore, serine proteases, which are present in high concentrations in *Bacillus*, can cleave peptide bonds on the carboxyl side of arginine and lysine residues, thereby enabling cell wall disruption [43].

This study showed that the culture suspension of *B. velezensis* JR4 greatly reduced postharvest rot and lesion size in carrots compared to the control group. Several studies have highlighted the effectiveness of using *B. velezensis* in controlling postharvest diseases. For instance, *B. velezensis* HG-8-2 strongly suppressed the incidence of anthracnose on pepper fruit caused by *Colletotrichum scovillei* by 61.1%., and lesions measured only 5.0 mm compared to 17.0 mm in the control group [44]. Another study found that treatments using a 50% of cell-free culture filtrate (CFCF) from *B. velezensis* BE1 significantly reduced the incidence and severity of gray mold in tomato fruit caused by *Botrytis cinerea* by 79.4% and 84%, respectively [45]. In this context, treating grapes with cell suspensions of *B. velezensis* P1 at 1.0 × 10^9^ CFU mL^− 1^ reduced the growth of *B. cinerea*, *C. acutatum*, *Aspergillus niger*, and *A. westerdijkiae* [46].

The phenylpropanoid pathway, also known as the phenolic metabolism pathway, plays a key role in plant defense against pathogens by inducing the production of secondary metabolites, such as total phenols, flavonoids, coumarin, lignin, and anthocyanins [47]. Phenolic compounds are substantial indicators of plant resistance and have the capacity to transform into quinones, which are highly toxic to plant pathogens. These substances accumulate during the early stages of infection, reinforcing the cell wall and preventing pathogen invasion [48]. Flavonoids are a class of phytoalexins found in plants that can inhibit the cellulases and pectinases produced by pathogens, helping prevent pathogens from penetrating the plant cell wall and strengthening the plant’s resistance [49]. In this study, *B. velezensis* JR4 consistently exhibited higher total phenolic and flavonoid content in carrot fruit treated with a bacterial suspension than in the control.

The cell membrane plays a key role in maintaining the integrity of vital cellular components, such as proteins, sugars, lipids, and nucleic acids. It also helps maintain the osmotic balance between the intracellular and extracellular environments. Initially composed of phospholipids and membrane proteins, the integrity of the cell membrane is critical for survival [43]. The permeability of a membrane can indicate how much a cell has aged or been damaged. When the plasma membrane is compromised, essential materials can leak out, which may eventually cause cell death [50]. In this study, there was a slight increase in conductivity after treatment with the bacterial suspension, in contrast to the significant rise observed in the control group. Another effect linked to oxidative stress relates to MDA content. MDA, a key byproduct of membrane lipid peroxidation, is commonly used as a biomarker of oxidative stress and cell damage [32]. Higher MDA levels indicate worsening cell membrane injury. The current results showed that carrots treated with the JR4 bacterial suspension had a lower MDA content than those in the control group. This may help to enhance their resistance to white mold after harvest. Additionally, an extreme concentration of reactive oxygen species (ROS), such as H_2_O_2_ and O_2_^−^, causes oxidative damage by destroying the structure and function of cell membranes. This disruption interferes with physiological processes and cell metabolism, as well as enhancing senescence. Therefore, maintaining redox homeostasis is crucial for the cells [51]. This study’s results indicate that treatment with the bacterial suspension of strain JR4 reduced H_2_O_2_ levels and O_2_^−^ content in postharvest carrots. These results suggest improved redox homeostasis and a lower ROS concentration.

Numerous studies have demonstrated the importance of activating defense-related enzyme activities, such as POD, PPO, and PAL, in disease resistance in fruits [45, 50]. This study also found that the activities of POD, PPO, and PAL were enhanced by bacterial suspensions of *B. velezensis* JR4 in carrot fruit, indicating that strain JR4 may contribute to triggering defense responses in fruit. In addition, the expression levels of the POD, PPO, and PAL genes were higher in carrot fruit treated with the suspension of *B. velezensis* JR4 than in the control group. These results demonstrate the close connection between defense-related gene expression, enzyme activity, and their key role in promoting fruit resistance. They also suggest that increased expression of these enzymes may improve disease resistance and reduce the occurrence of postharvest fungal diseases in carrot fruit.

According to the cytotoxicity tests in the current study, the cell-free culture filtrate of *B. velezensis* JR4 exhibited no or only low toxicity on both WI-38 and Vero cells. In the same way, bacteriocins from *Bacillus* strains have been found to have varying cytotoxic effects on mammalian cell lines. For example, the bacteriocin produced by *B. coagulans* BDU3 displayed minimal toxicity toward HEK 293 human embryonic kidney cells [52]. The IC_50_ of the *B. licheniformis* P40 bacteriocin against Vero cells was 0.30 µg mL^− 1^ [53]. Meanwhile, a bacteriocin from *B. velezensis* BUU004 appears promising as a safe food preservative, according to Butkhot et al. [54]. Based on the criteria recommended by EFSA in 2014 [55], *B. velezensis* BUU004 could receive QPS status. This highlights that *B. velezensis* BUU004 has essential biosafety characteristics, poses no harm to human health, and is safe for human use.

Although *B. velezensis* shows great promise as an alternative to conventional pesticides in sustainable agricultural practices and biotechnological applications, there are still rigorous challenges and limitations to be addressed for its successful implementation. These include stability and viability during storage and application, competition with plant microbiomes, performance comparisons with commercial fungicides, the production of bioactive metabolites in various quantities, safety assessments, and compliance with regulatory standards. Ongoing advances in molecular research are expected to improve our understanding of the functions of *B. velezensis*, which could make it more practical for application.

## Conclusions

This study established *B. velezensis* JR4 as an effective biocontrol agent against *S. sclerotiorum*, demonstrating strong suppression in both in vitro and in vivo conditions. The biocontrol mechanism of JR4 may be attributed to host defense priming through the induction of defense-related enzymes (POD, PPO, and PAL), increased activity of related genes, and the accumulation of phenolic compounds, as well as the reduction of oxidative indicators. The cell-free culture filtrate of the strain exhibited minimal to no toxicity to WI-38 and Vero cells. The results confirmed that JR4 represents a promising candidate for future development as a biological control agent.

## Data Availability

All data generated or analyzed during this study are included in this article. The sequences utilized in this study have been deposited in GenBank under accession number PV973319.1.
